# Iberian pig mesenchymal stem/stromal cells from dermal skin, abdominal and subcutaneous adipose tissues, and peripheral blood: in vitro characterization and migratory properties in inflammation

**DOI:** 10.1186/s13287-018-0933-y

**Published:** 2018-07-04

**Authors:** Alexandra Calle, Clara Barrajón-Masa, Ernesto Gómez-Fidalgo, Mercedes Martín-Lluch, Paloma Cruz-Vigo, Raúl Sánchez-Sánchez, Miguel Ángel Ramírez

**Affiliations:** Departamento de Reproducción Animal, Instituto Nacional de Investigación y Tecnología Agraria y Alimentaria, Avenida Puerta de Hierro 12, local 10, 28040 Madrid, Spain

**Keywords:** Mesenchymal stem/stromal cells, Iberian pig, Cell migration, Inflammation

## Abstract

**Background:**

Recently, the capacity of mesenchymal stem/stromal cells (MSCs) to migrate into damaged tissues has been reported. For MSCs to be a promising tool for tissue engineering and cell and gene therapy, it is essential to know their migration ability according to their tissue of origin. However, little is known about the molecular mechanisms regulating porcine MSC chemotaxis. The aim of this study was to examine the migratory properties in an inflammatory environment of porcine MSC lines from different tissue origins: subcutaneous adipose tissue (SCA-MSCs), abdominal adipose tissue (AA-MSCs), dermal skin tissue (DS-MSCs) and peripheral blood (PB-MSCs).

**Methods:**

SCA-MSCs, AA-MSCs, DS-MSCs and PB-MSCs were isolated and analyzed in terms of morphological features, alkaline phosphatase activity, expression of cell surface and intracellular markers of pluripotency, proliferation, in vitro chondrogenic, osteogenic and adipogenic differentiation capacities, as well as their ability to migrate in response to inflammatory cytokines.

**Results:**

SCA-MSCs, AA-MSCs, DS-MSCs and PB-MSCs were isolated and showed plastic adhesion with a fibroblast-like morphology. All MSC lines were positive for CD44, CD105, CD90 and vimentin, characteristic markers of MSCs. The cytokeratin marker was also detected in DS-MSCs. No expression of MHCII or CD34 was detected in any of the four types of MSC. In terms of pluripotency features, all MSC lines expressed POU5F1 and showed alkaline phosphatase activity. SCA-MSCs had a higher growth rate compared to the rest of the cell lines, while the AA-MSC cell line had a longer population doubling time. All MSC lines cultured under adipogenic, chondrogenic and osteogenic conditions showed differentiation capacity to the previously mentioned mesodermal lineages. All MSC lines showed migration ability in an agarose drop assay. DS-MSCs migrated greater distances than the rest of the cell lines both in nonstimulated conditions and in the presence of the inflammatory cytokines TNF-α and IL-1β. SCA-MSCs and DS-MSCs increased their migration capacity in the presence of IL-1β as compared to PBS control.

**Conclusions:**

This study describes the isolation and characterization of porcine cell lines from different tissue origin, with clear MSC properties. We show for the first time a comparative study of the migration capacity induced by inflammatory mediators of porcine MSCs of different tissue origin.

## Background

Mesenchymal progenitors are a group of adult multipotential stem cells that were first characterized in 1976 by Friedenstein, who isolated them from bone marrow and described them as adherent cells with fibroblastoid morphology, able to differentiate into cells of mesodermal origin such as osteocytes, chondrocytes or adipocytes [1]. Thus, mesenchymal stem cells, also referred to as multipotent stromal cells or mesenchymal stromal cells (MSCs) [2, 3], are multipotent cells with significant clinical importance because of their applicability in cell therapy for regenerative medicine and tissue engineering [4]. In addition, various studies have demonstrated that MSCs are strongly immunosuppressive both in vitro and in vivo [5–10], being able to reduce graft-versus-host disease associated with allografts and xenografts [11].

In 2006, with the aim of standardization, the International Society for Cellular Therapy proposed three criteria to define the minimal characteristics of MSCs [12]: when maintained in standard culture conditions using tissue culture flasks, they should display plastic adherence; more than 95% of the MSC population should express specific markers such as CD105, CD73 and CD90, and be negative for CD45, CD34, CD14 or CD11b, CD79α or CD19 and HLA class II; and they should be able to differentiate to osteoblasts, adipocytes or chondroblasts in vitro under standard differentiating conditions as demonstrated by specific staining of in vitro cell cultures. The use of MSCs in regenerative medicine in human and animals is increasing as their characteristics of self-renewal, proliferative capacity and differentiation potential are becoming better controlled. In addition, the ISCT criteria do not guarantee the purification of homogeneous populations of MSCs, and in fact the isolation of MSCs with ISCT criteria produces nonclonal and heterogeneous cultures of stromal cells, stem cells, progenitor cells and differentiated cells [13].

Previously, many experimental animals such as mouse, rat, and rabbit have been tested as models for clinical applications; however, the importance of pigs has been highlighted as the best experimental model, based on the similarities of porcine organ physiology with human beings [14]. Pigs are currently the animal model of choice for evaluation of stem cell-based therapy, regenerative medicine and transplantation [15]. Within pigs, there are genetic differences among pig subspecies [16] and Iberian pigs are at risk for obesity and cardiometabolic diseases in case of an excess of nutrients, a risk reported either at juvenile development or at adulthood [17]. Thus, Iberian breeding sows are highly sensitive to nutritional and metabolic changes, much more than lean breeds [18]. For all these reasons according to its similarity with human obesity and metabolic diseases, the Iberian pig has been proven particularly valuable as a biomedical-research animal model for human investigation. Besides, in terms of animal production, the Iberian pig stimulates important economic interest in the ambit of livestock. Indeed, the Iberian pig is known worldwide for the production of a unique highly priced drycured product, Iberian ham, with a unique taste due to its abundance in intramuscular fat. In fact, the Iberian pig has a high potential for fat accumulation under its skin and among the muscular fibers [19]. Generation of specific porcine cell lines will help in a variety of experimental research and in understanding stem cell xenotransplantation safety in an excellent animal model.

MSCs have been described in different porcine tissues, exhibiting the aforementioned stem cell properties like plastic adherence, multilineage differentiation capacity, expression of MSC markers and pluripotent genes. It is clearly evident that postnatal organs and tissues serve as good MSC sources; however, each source of MSCs has a different extent of differentiation potential and expression of a different combination of stem cell-related markers and other important features like high proliferation, immunomodulation and xenotransplantation ability. Therefore, suitable MSCs should be carefully validated for cell-based therapies before clinical application.

One of the most remarkable but least understood findings is the ability of human MSCs to migrate from bone marrow or peripheral blood into damaged tissues. Transplantation experiments in animals and patients demonstrated that MSCs migrate to sites of injury, where they enhance wound healing [20], support tissue regeneration following myocardial infarction [21], home to and promote the restoration of the bone marrow microenvironment after damage by myeloablative chemotherapy [22] or help to overcome the molecular defect in children with osteogenesis imperfecta [23]. Although Almalki et al. [24] have recently reported porcine abdominal adipose tissue MSC (AA-MSC) migration ability mediated by cytokines, little is known about the molecular mechanisms regulating cell movement and relocalization in porcine MSCs. For MSCs to be a promising tool for tissue engineering and cell and gene therapy strategies, it is essential to know their migration ability according to their tissue of origin.

The most obvious disadvantages of the majority of tissular sources of MSCs described so far are the invasiveness of the harvesting procedure. An excellent alternative source of cells is blood, such as umbilical cord blood collected at birth or peripheral blood (PB) from adult animals. Given that such blood samples can be readily taken in a sterile manner, they may provide a readily accessible source of autologous MSCs for regenerative therapies. In order to standardize the promising results of such therapy, it is essential that well-characterized and homogeneous MSC populations be used. Currently, MSCs have been isolated from peripheral blood (PB-MSCs) of human, mice, sheep, horse, dog, cat, rat, rabbit and pig [7, 25–30]. Despite this trend, basic information regarding pig PB-MSCs is still limited.

## Methods

### Isolation, culture and karyotyping analysis of MSCs

Abdominal adipose tissue, subcutaneous adipose tissue and dermal skin were obtained post mortem from an adult Iberian boar. Previously, a blood sample was harvested from the jugular vein (5 ml) using heparin vacutainer tubes.

The collected samples for isolation and culture of AA-MSCs, SCA-MSCs and DS-MSCs were rinsed several times with water and washed three times with Hank’s Balanced Salt Solution (HBSS) supplemented with 500 U/ml penicillin, 500 mg/ml streptomycin and 0.1% bovine serum albumin (BSA) (Merck KGaA, Darmstadt, Germany).

Adipose and dermal skin tissues were minced using sterile scissors to enhance collagenase type II (Gibco by Life Technologies, Grand Island, NY, USA) action. Minced tissues were incubated in a collagenase type II solution—HBSS supplemented with 0.05% collagenase type II, 0.1% BSA and 30 nM CaCl_2_—during 45 min at 37 °C, shaking gently every 5 min. Thereafter, a volume of culture medium—Dulbecco’s modified Eagle’s medium low glucose (DMEM-LG) (Hyclone Laboratories, UT, USA), supplemented with 15% fetal calf serum (PAA Laboratories, Austria), 2% nonessential amino acids and antibiotics (100 U/ml penicillin, 100 mg/ml streptomycin)—was added to block the action of collagenase and the obtained suspension centrifuged at 300 × *g* for 5 min.

The resulting pellets were resuspended in culture medium and plated in a 100-mm^2^ tissue culture dish (JetBiofil, Guangzhou, China) and incubated in an atmosphere of humidified air and 5% CO_2_ at 37 °C. Culture medium was changed every 48–72 h.

Isolated colonies of putative MSCs were apparent after 6–8 days in culture and were maintained in growth medium until ~ 75% confluence.

The cells were then treated with 0.05% trypsin–EDTA (T/E) and further cultured for subsequent passage in 100-mm^2^ dishes at 50,000 cells/cm^2^.

To isolate peripheral blood-derived mononuclear cells, phosphate buffered saline (PBS) 1:1 diluted blood (5 ml) was layered onto 10 ml Biocoll separating solution (Biochrom AG, Germany) in a 100-ml tube and centrifuged at 1600 × *g* for 20 min. The mononuclear cells were collected from the interphase, washed twice with PBS by centrifugation at 3000 × *g* for 15 min and then suspended in DMEM-LG supplemented with 10% FCS, 2 mM glutamine, 1 mM MEM nonessential amino acid solution and antibiotics (100 U/ml penicillin, 100 mg/ml streptomycin). Cells obtained from each 30 ml of blood were seeded onto a 100-mm^2^ tissue culture dish and incubated in an atmosphere of humidified air and 5% CO_2_ at 37 °C. Nonadherent cells were removed by washing twice with PBS after 48 h of incubation and fresh complete medium was then added to the dishes. Thereafter, the medium was changed every 48–72 h and split at ~ 75% confluence as before.

The MSC chromosome preparation was carried out following the procedures of Rodríguez et al. [31] with minor modifications. Briefly, cells were incubated with 0.1 μg/ml colcemid (Gibco) for 60 min in a humidified incubator (5% CO_2_, 37 °C) and then detached. The pelleted cells were incubated in 5 ml of hypotonic solution (0.057 M KCl) for 10 min at room temperature followed by fixation with methanol/glacial acetic acid (3:1) solution. Fixed cells were dropped on wet slides and air-dried overnight at 60 °C to obtain a GTL-banding chromosome pattern. Leishman solution for GTL-banding was carried out and metaphases were fully karyotyped under a Nikon Eclipse E400 microscope. Images were then captured with a digital camera IAI® Progressive scan using Cytovision Genus® software.

### Inmunocytochemical analysis by flow cytometry

Surface, cytoplasmic and nuclear cell antigens were examined by flow cytometry using a Cell Lab Quanta SC system from Beckman Coulter.

Cell cultures at 80–90% confluence were detached using T/E solution, collected and fixed with 4% paraformaldehyde for 10 min and subsequently washed twice with PBS.

For analysis of the expression of vimentin (clone LN-6; Sigma-Aldrich), cytokeratin (Clone C-11; Sigma-Aldrich) (cytoplasmic proteins) and POU5F1 (rabbit polyclonal; Biorbyt) (a nuclear protein), cell permeabilization was performed by incubation with 0.3–0.5% Triton X-100 for 10 min and washing with PBS. Nonspecific binding of the antibodies was blocked with TNB-blocking solution during 30 min at 37 °C.

Appropriate dilutions, provided by manufacturers, of primary antibodies against the markers commonly used to define MSCs—vimentin (clone LN-6; Sigma-Aldrich), CD44 (clone IM7; Bio-rad), CD105 (clone MEM-229; Abcam) and CD90 (clone 5E10; Abcam) as positive markers, cytokeratin, CD34 (rabbit polyclonal; Biorbyt) and MHCII (clone CVS20; Bio-Rad) as negative markers and POU5F1 as a pluripotency marker—were added to the cells and incubated overnight at 4 °C. Cells were then stained with the appropriated Alexa fluor 488-conjugated secondary antibodies (Jackson InmunoResearch Laboratories, West Grove, PA, USA). Negative control samples were obtained by omission of the primary antibody. Analysis of the samples was performed with Cell Lab Quanta SC system from Beckman Coulter using Flow-Jo X SOFTWARE® version 10.0.7r2.

### Alkaline phosphatase activity

AA-MSC, SCA-MSC, DS-MSC and PB-MSC lines at passages 10–15 were grown on 35-mm dishes (JetBiofil, Guangzhou, China) for 2 weeks. Cells were washed twice with PBS and fixed with a solution of 4% paraformaldehyde during 10 min at room temperature. Paraformaldehyde was aspirated and the plates were washed twice with distilled water and covered with Solution B (1 ml of Solution A (Fast Red 1 mg/ml), 1.6 μl of Napthol AS-mx phosphate and 40 μl Tris–HCl 1 M, pH 8.6) during 10–15 min at room temperature in the dark. Solution B was finally removed and the cells were washed twice with PBS and covered with PBS to prevent drying. The colonies were examined for appearance of pink/red coloration indicating alkaline phosphatase (AP) activity. The stained colonies were imaged using an inverted Nikon Diaphot phase-contrast microscope coupled to a Jenoptik ProgRes CT1 digital camera. Images were captured using ProgRes capture pro software version 2.7 (Jenoptik Laser, Optic Systeme GmbH).

### Cell proliferation measurement

The different mesenchymal cell lines at passages 9–11 were seeded at 2 × 10^5^ cells per 60-mm tissue culture plates (JetBiofil, Guangzhou, China). The culture medium was changed every 2 days. At each time point a duplicate of plates were detached by tripsinization and counted using a Bürker counting chamber (Paul Marienfeld GmbH & Co., Lauda-Königshofen, Germany). Then, 20 μl of cell suspension was placed in both sides of the chamber and viewed using 100× magnification under an inverted Nikon Diaphot phase-contrast microscope coupled to a Jenoptik ProgRes CT1 digital camera. Images were captured using ProgRes capture pro software version 2.7 (Jenoptik Laser, Optic Systeme GmbH).

A total of 5 × 1 mm^2^ squares per sample were analyzed and the number of cells per milliliter was determined according to the equation:

$$ \mathrm{Number}\ \mathrm{of}\ \mathrm{cells}\ \mathrm{in}\ 1\ \mathrm{ml}=\mathrm{N}/\mathrm{Z}\times \mathrm{dilution}\times {10}^4, $$

where *N* = the whole number of cells counted and *Z* = the number of counted squares.

The cell population doubling time (PDT) was calculated using Roth V. 2006 Doubling Time Computing (available from http://www.doubling-time.com/compute.php).

### In vitro differentiation potential assay

AA-MSC, SCA-MSC, DS-MSC and PB-MSC lines at passages 10–15 were grown until 90% confluence on a 12-well/24-well multidish (JetBiofil, Guangzhou, China).

For adipogenic differentiation, the StemPro® Adipogenesis Differentiation Kit (Thermo Fisher Scientific, Rockford, IL, USA) was used according to the manufacturer’s instructions. Differentiating media were changed every 2–3 days for 14 days. Simultaneously, control cells were cultured in standard conditions. Cells were then fixed in 4% paraformaldehyde solution for 10–15 min. After fixation, cells were incubated for 5 min in 60% isopropanol and stained with Oil red O (Merck KGaA, Darmstadt, Germany) solution to visualize the accumulation of red lipid droplets. Cells were photographed using a Nikon Diaphot light microscope coupled to a Canon EOS 500D digital camera.

For osteogenic differentiation, the StemPro® Osteogenesis Differentiation Kit (Thermo Fisher Scientific) was used according to the manufacturer’s instructions. Differentiating media were changed every 3–4 days for 21 days. Simultaneously, control cells were cultured in standard conditions. Cells were then fixed in 4% paraformaldehyde solution for 30 min. After fixation, cells were incubated for 2–3 min in 2% Alizarin Red S solution (pH 4.2) to visualize the calcium deposits.

For chondrogenic differentiation, the StemPro® Chondrogenesis Differentiation Kit (Thermo Fisher Scientific) was used according to the manufacturer’s instructions. Differentiating media were changed every 2–3 days for 14 days. Simultaneously, control cells were cultured in standard conditions. Cells were then fixed in 4% paraformaldehyde solution for 30 min. After fixation, cells were incubated for 30 min with 1% Alcian Blue solution prepared in 0.1 N HCl. Blue staining was corresponding with proteoglycans synthetized by chondrocytes. Cells under ostegenesis and chondrogenesis differentiation conditions were photographed using a Motic SMZ-171 stereomicroscope coupled to a Moticam BTU8 digital camera.

### Cell migration measurement: agarose spot assay

The cell migration measurement by agarose spot assay was carried out following the procedures of Wiggins and Rappoport [32] with minor modifications. Briefly, PBS–0.5% agarose solution was heated on a water bath until boiling to facilitate complete dissolution. When the temperature cooled down to 40 °C, 90 μl of agarose solution was pipetted into a 1.5-ml Eppendorf tube containing 10 μl of PBS or PBS supplemented with TNF-α or IL-1β for a final concentration of 6 nM [33]. Then, 5-μl spots of agarose-containing PBS, TNF-α or IL-1β were pipetted onto six-well plates (JetBiofil, Guangzhou, China), 16 drops per well, 12 drops per MSC line, and allowed to cool for 15 min at 4 °C. At this point, cells that had been treated with C-Mitomycin 1 μg/ml overnight (Merck KGaA, Darmstadt, Germany) to avoid cellular duplication were plated onto spot-containing dishes in the presence of culture media. Imaging was performed at 24 and 48 h using a Motic SMZ-171 stereomicroscope coupled to a Moticam BTU8 digital camera and Motic Image Plus software version 2.0 (Motic China Group Co., Ltd). Motile cells penetrated the agarose spot. The longest straight distance from the border of the spot was analyzed for each cell using Image J.

### Statistical analysis

Statistical analysis was performed using GraphPad Prism 6 (GraphPad Software, La Jolla, CA, USA). One-way ANOVA for multiple comparisons by Fisher’s LSD tests was used for cell proliferation and doubling time. Two-way ANOVA for multiple comparisons by Fisher’s LSD tests was used for cell migration. Values are expressed as mean ± standard error of the mean (SEM). Differences were considered to be significant when *p* < 0.05.

## Results

### Morphological features and chromosomal stability

**Fig. 1:**
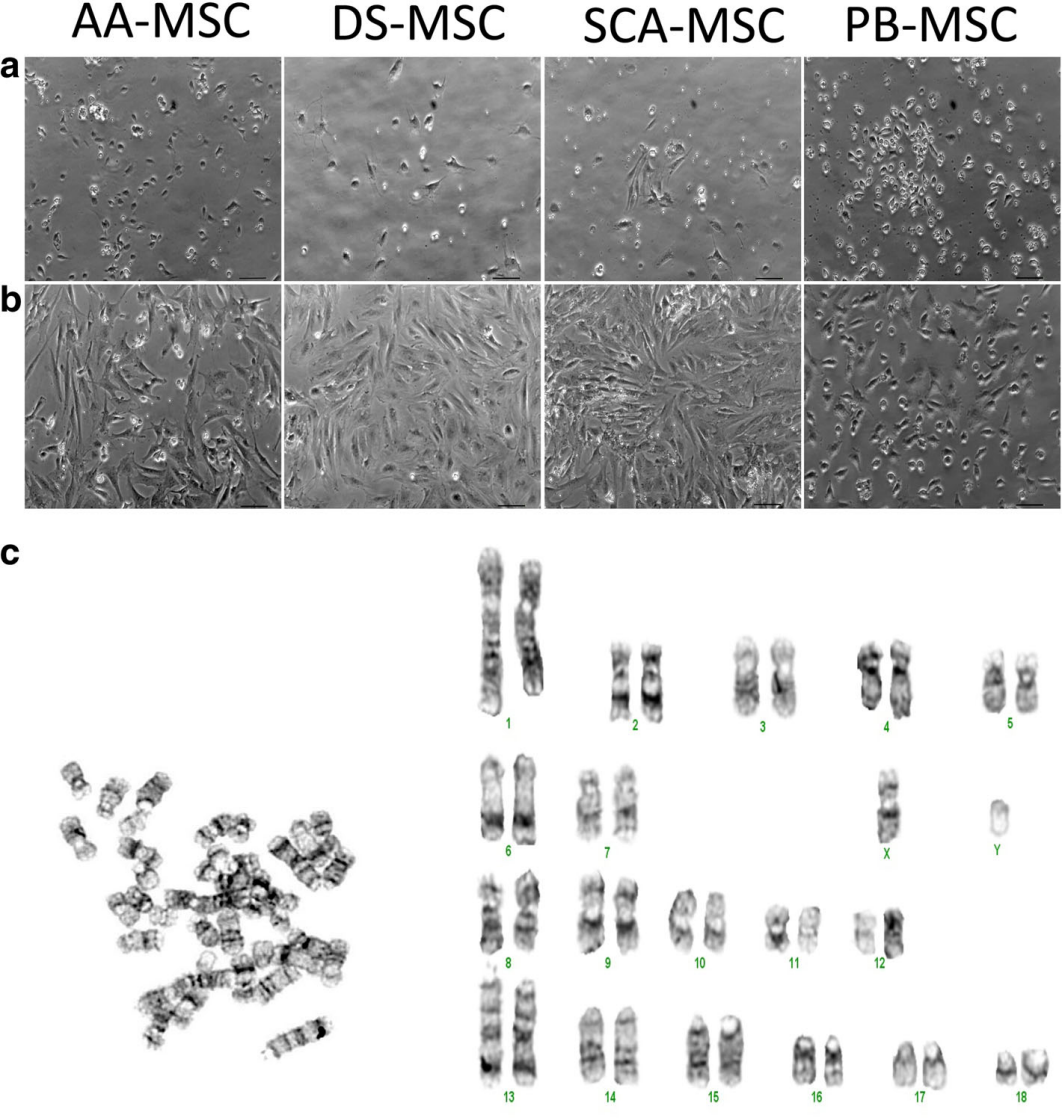
Morphology of MSCs at (**a**) passage 0 and 8 days of culture and (**b**) first passage and 13 days of culture. Phase-contrast images acquired with 100× magnification. Bars = 70 μm. (**c**) Representative P10 metaphase and karyotype. No chromosomal aberrations observed in AA-MSCs after long-term cultivation. AA-MSC abdominal adipose tissue mesenchymal stem/stromal cell, DS-MSC dermal skin tissue mesenchymal stem/stromal cell, PB-MSC peripheral blood mesenchymal stem/stromal cell, SCA-MSC subcutaneous adipose tissue mesenchymal stem/stromal cell

As shown in Fig. 1, we could successfully isolate MSCs from abdominal adipose tissue, subcutaneous adipose tissue, dermal skin and peripheral blood of an adult male Iberian pig. In primary culture, MSCs of all four sources adhered to the plastic surface of culture dishes, exhibiting a mixture of round, spindle or elongated shape morphologies (Fig. 1a). However, after the first cell passage, cells formed a homogeneous population of fibroblast-like adherent cells (Fig. 1b).

To analyze the chromosomal stability of MSCs during in vitro culture, the AA-MSC line expanded through 10 passages was used for GTL-banding. No chromosomal translocation, deletion or extra-chromosome was observed (Fig. 1c).

### Expression of cell surface, intracellular and pluripotency markers

**Fig. 2:**
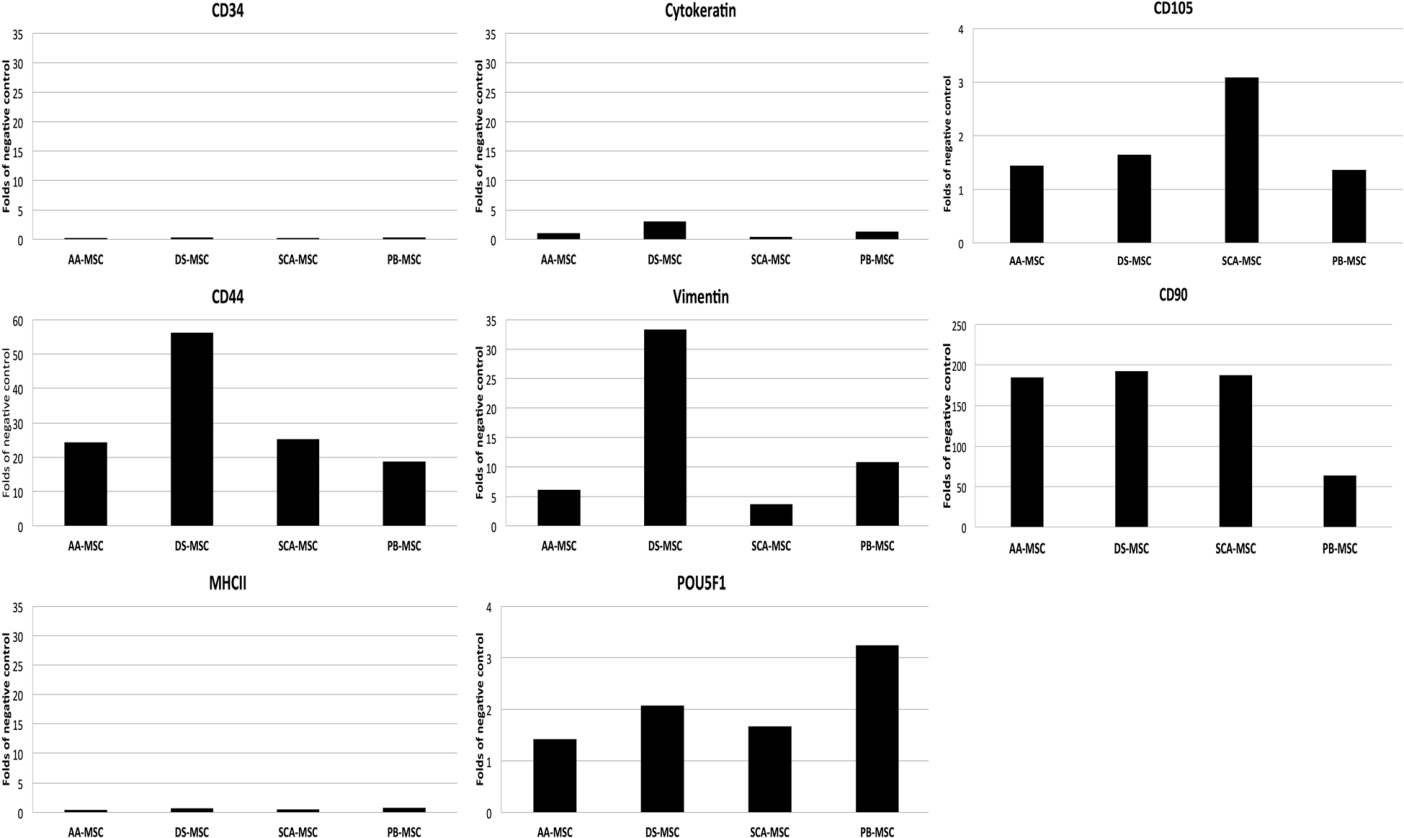
Analysis by flow cytometry of expression levels of cell surface markers CD34, CD44, CD105, CD90 and MHCII and intracellular markers cytokeratin, vimentin and POU5F1 in AA-MSCs, DS-MSCs, SCA-MSCs and PB-MSCs. Data correspond to mean fluorescence intensity (fold of negative control) for each sample. AA-MSC abdominal adipose tissue mesenchymal stem/stromal cell, DS-MSC dermal skin tissue mesenchymal stem/stromal cell, MHCII major histocompatibility complex II, PB-MSC peripheral blood mesenchymal stem/stromal cell, POU5F1 POU class 5 homeobox 1, SCA-MSC subcutaneous adipose tissue mesenchymal stem/stromal cell

Expression of MSC markers has been reported to differ in porcine MSCs from different tissue origin [34]. For further characterization of all four types of MSCs, some characteristic cell surface and intracellular markers were assessed by flow cytometry (Fig. 2). All cell types were positive for cell surface expression of CD44, CD105, CD90 and the cytoplasmic marker vimentin, characteristic of MSCs. Interestingly, the cytoplasmic marker cytokeratin, typically from epithelium of ectoderm and endoderm, commonly used as a negative marker of MSCs, could also be detected in DS-MSCs. No expression of immune-phenotype markers, such as MHCII or CD34, was detected in any of the four lines of MSCs (Fig. 2).

**Fig. 3:**
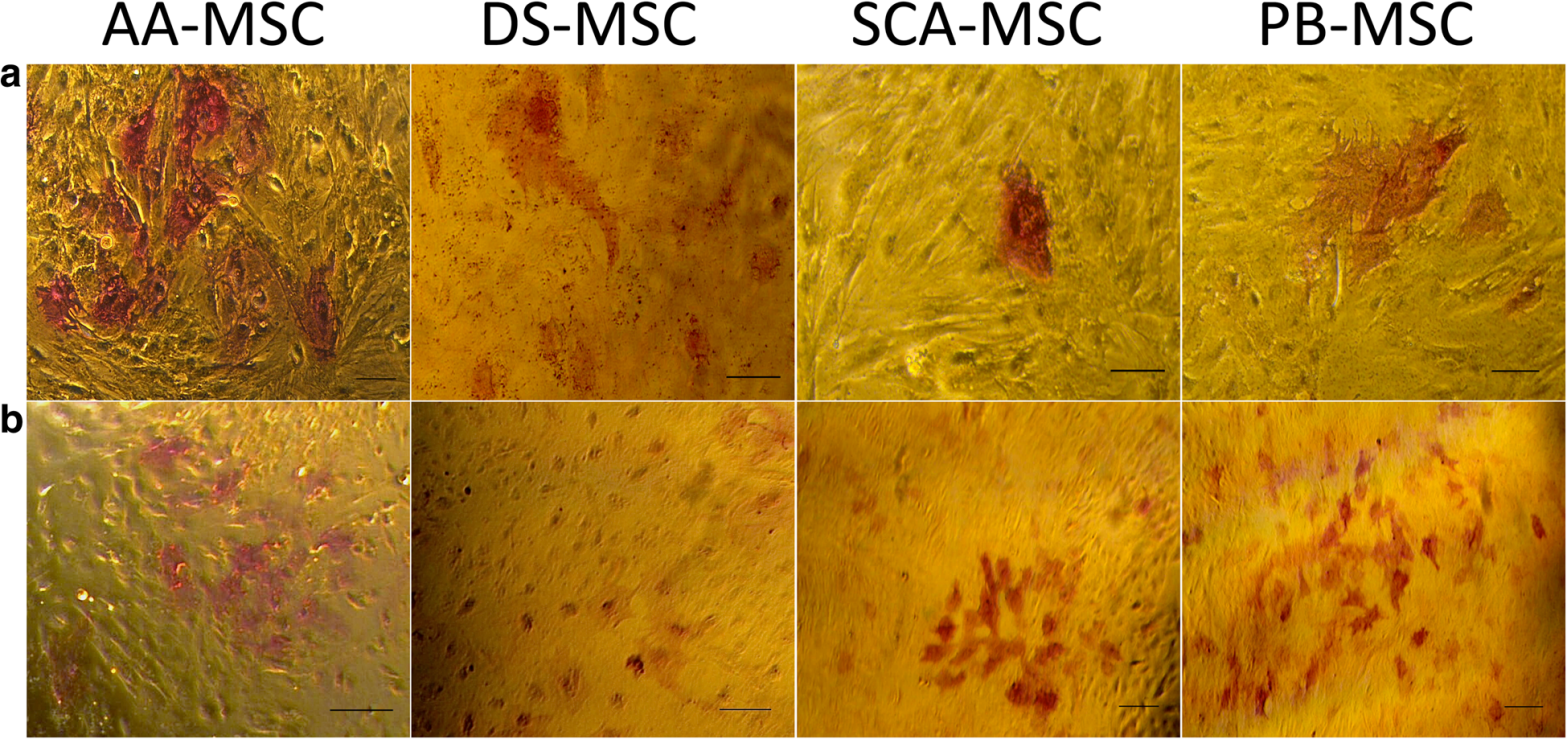
Analysis of alkaline phosphatase (AP) activity. Bright-field images obtained at 100× (**a**) or 32× (**b**) magnification, showing some red-stained cell groups after action of alkaline phosphatase on Fast Red in presence of Napthol AS-mx phosphate. Bars = 70 μm (top panels) and 150 μm (bottom panels). AA-MSC abdominal adipose tissue mesenchymal stem/stromal cell, DS-MSC dermal skin tissue mesenchymal stem/stromal cell, PB-MSC peripheral blood mesenchymal stem/stromal cell, SCA-MSC subcutaneous adipose tissue mesenchymal stem/stromal cell

MSC lines were analyzed for pluripotency features. All MSC lines were positive for the nuclear marker POU5F1 (Fig. 2), and stained positive for alkaline phosphatase (Fig. 3). The lowest level of alkaline phosphatase activity was observed in DS-MSCs.

### Proliferation capacity

**Fig. 4:**
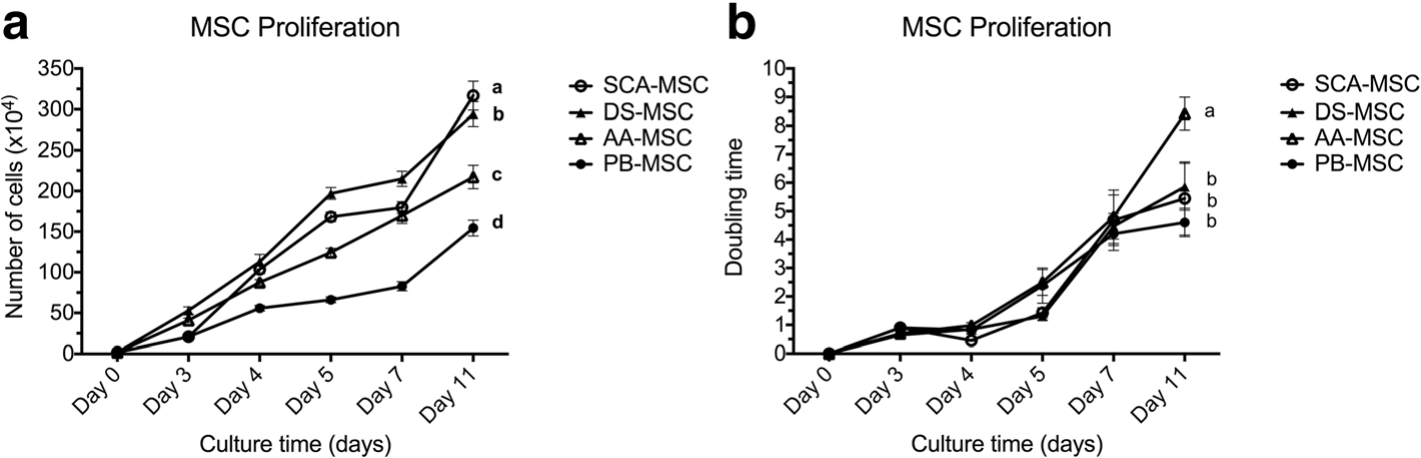
In vitro proliferation of MSCs. (**a**) Absolute number of cells/dish (mean ± SD). (**b**) Doubling time of each MSC line (mean ± SD). Different lowercase letters indicate significant differences (*p* < 0.05). AA-MSC abdominal adipose tissue mesenchymal stem/stromal cell, DS-MSC dermal skin tissue mesenchymal stem/stromal cell, MSC mesenchymal stem/stromal cell, PB-MSC peripheral blood mesenchymal stem/stromal cell, SCA-MSC subcutaneous adipose tissue mesenchymal stem/stromal cell

To analyze the cell proliferation capacity of MSCs, the number of cells/dish was counted for each cell line at days 3, 4, 5, 7 and 11, starting in all cases from an initial seeding of 2 × 10^5^ cells. As shown in Fig. 4A the number of cells increased for all cell lines along the entire assay. On day 11, the 60-mm culture plate contained the following total number of cells: for the most proliferative line SCA-MSCs, 316.8 × 10^4^ ± 30.9 × 10^4^ cells; for DS-MSCs, 294.3 × 10^4^ ± 47.4 × 10^4^ cells; for AA-MSCs, 217.2 × 10^4^ ± 45.3 × 10 ^4^ cells; while PB-MSCs, with a significantly lower proliferation rate throughout the experiment, presented 154.5 × 10^4^ ± 30.9 × 10^4^ cells.

Figure 4b shows the proliferation rate of MSCs in terms of the population doubling time (PDT). On day 11, AA-MSCs showed a significantly higher PDT (8.4 ± 1.4 days) than the rest of the MSC lines (DS-MSCs 5.9 ± 1.8 days, SCA-MSCs 5.4 ± 3.6 days and PB-MSCs 4.6 ± 1.5 days).

### In vitro differentiation of MSCs

**Fig. 5:**
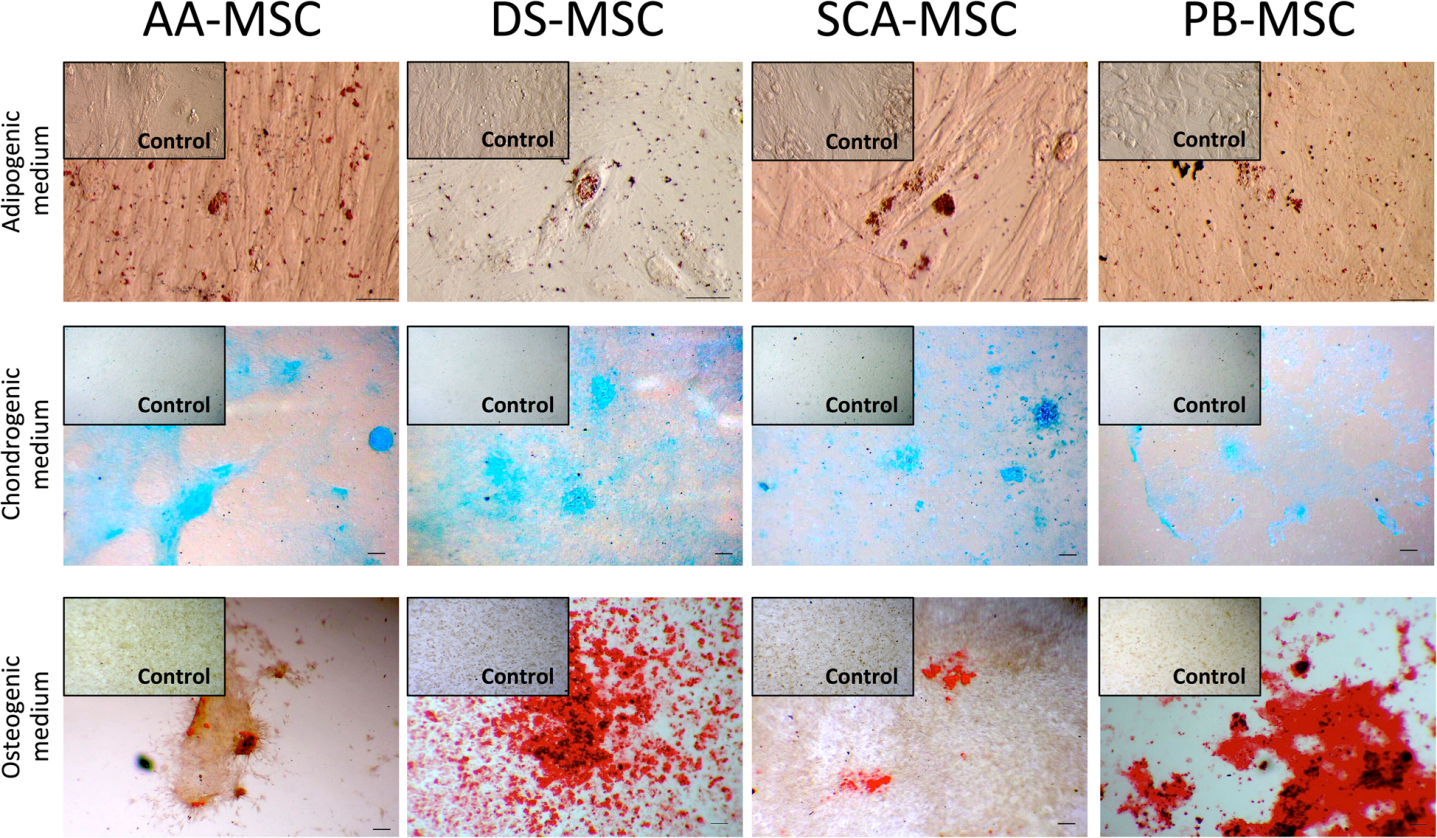
In vitro differentiation of MSCs to different lineages. Images show Oil red O staining of lipid droplets in cells cultured in basal medium (Control) or in adipogenic differentiation medium (top panel); Alcian blue staining of acidic proteoglycan in cells cultured in basal medium (Control) or in chondrogenic differentiation medium (middle panels); and Alizarin Red S staining of calcium deposits in cells cultured in basal medium (Control) or in osteogenic differentiation medium (bottom panels). Bright-field images acquired with 200× magnification (bars = 70 μm) for top panels and 3× magnification (bars = 150 μm) for middle and bottom panels. AA-MSC abdominal adipose tissue mesenchymal stem/stromal cell, DS-MSC dermal skin tissue mesenchymal stem/stromal cell, PB-MSC peripheral blood mesenchymal stem/stromal cell, SCA-MSC subcutaneous adipose tissue mesenchymal stem/stromal cell

As shown in Fig. 5, all MSC lines cultured under adipogenic or osteogenic conditions presented cytoplasmic lipid droplets or distinctive calcium deposits, respectively. A comparable amount of cytoplasmic lipid droplets was observed in all MSCs while the staining pattern of calcium deposits was strongest in DS-MSCs and PB-MSCs, indicating a high potential for differentiation of these lines. Cells cultured under chondrogenic conditions showed the presence of acidic proteoglycan that was demonstrated at monolayer cells by Alcian blue staining. Besides, AA-MSCs presented stained nodules typical from cartilaginous tissue phenotype.

### Migration ability of MSC lines

**Fig. 6:**
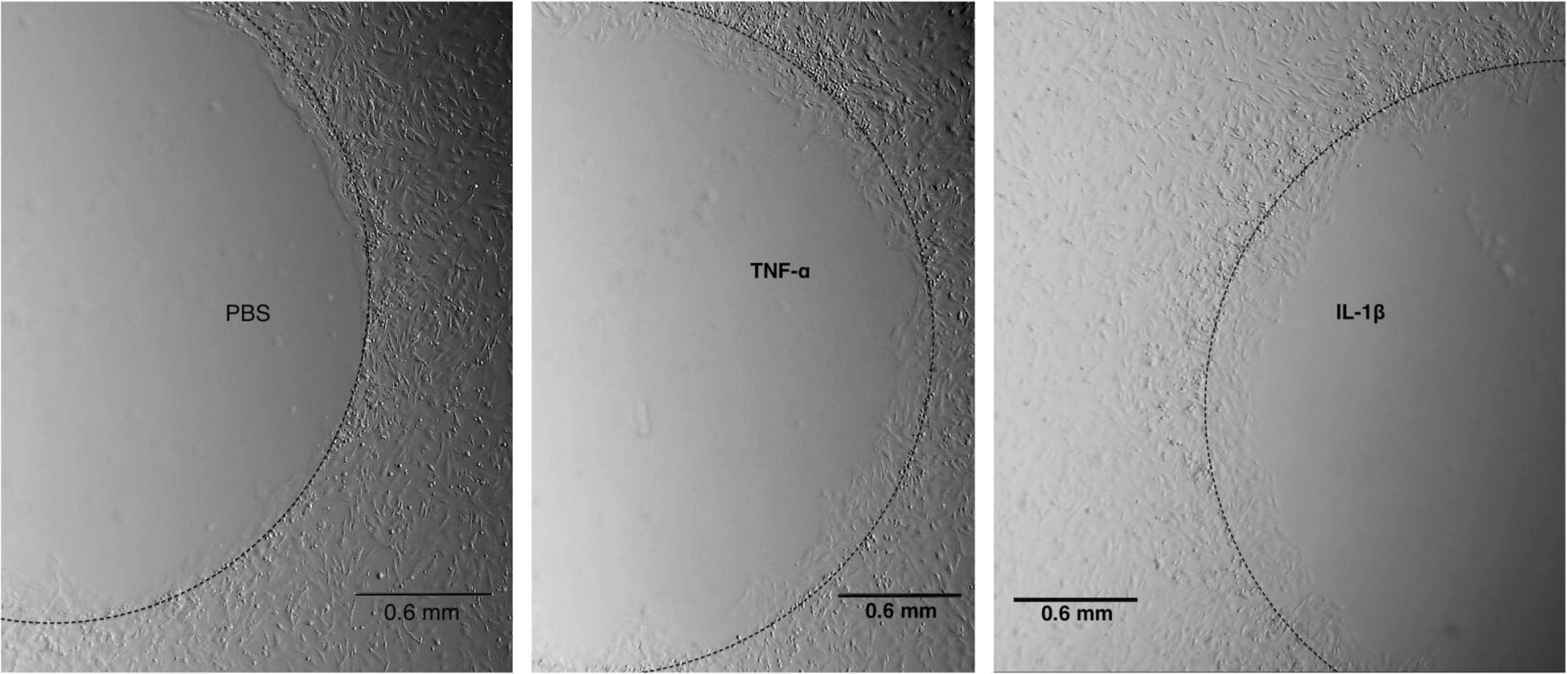
Representative images of AA-MSC migration assay into PBS, TNF-α or IL-1β-agarose spot after 48 h. Images obtained in a light stereomicroscope at 20× magnification. IL-1β interleukin-1β, PBS phosphate buffered saline, TNF-α tumor necrosis factor alpha

**Fig. 7:**
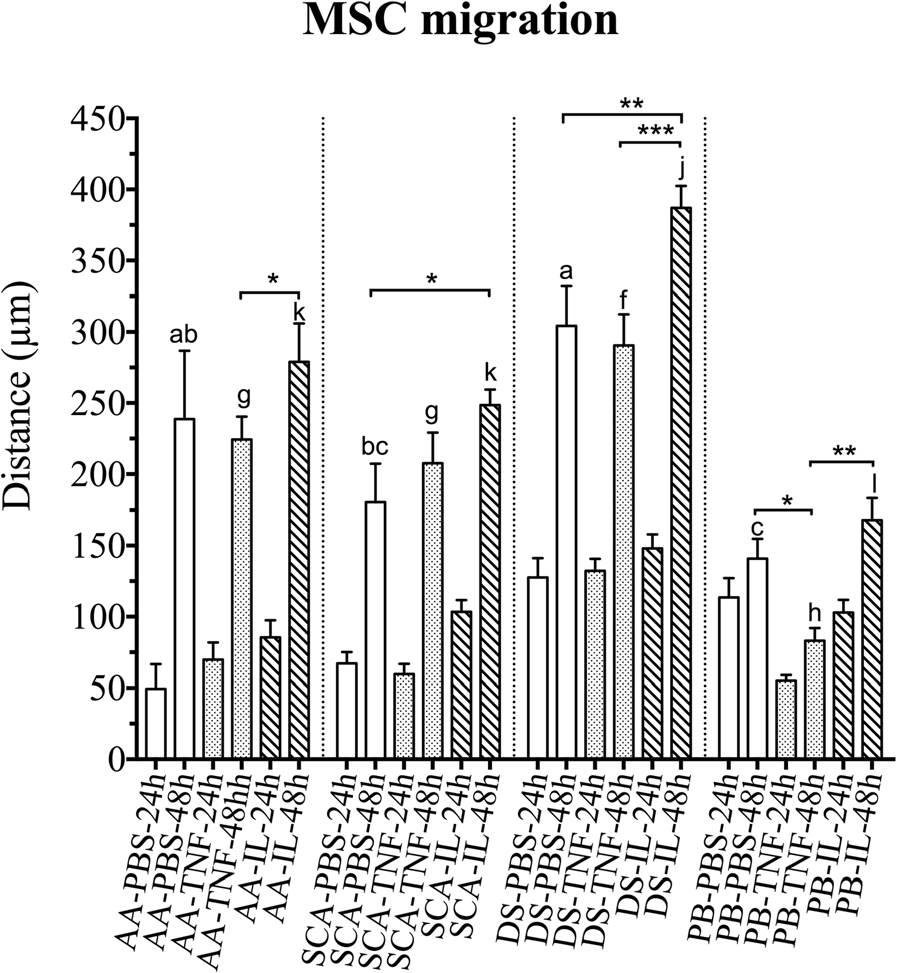
Migration analysis in agarose spot assay. Distance migrated from border of agarose spot measured in two independent experiments for AA-MSCs, SCA-MSCs, DS-MSCs and PB-MSCs at 48 h (mean ± SD). Different lowercase letters indicate significant differences (*p* < 0.05 for MSC migration mediated by PBS (a, b, c) and TNF-α (f, g, h); *p* < 0.005 for MSC migration mediated by IL-1β (j, k, l)). *p < 0.05; **p < 0.005; ***p < 0.0005. AA abdominal adipose tissue, DS dermal skin tissue, IL interleukin, MSC mesenchymal stem/stromal cell, PB peripheral blood, PBS phosphate buffered saline, SCA subcutaneous adipose tissue, TNF tumor necrosis factor

Assessment of the invasion capacity of all MSC lines was performed using the agarose spot assay [32] with minor modifications. This assay allows the measurement of cell invasion by analyzing the crawling of the cells underneath an agarose gel on a planar surface (Fig. 6). All MSC lines showed migration capacity in the agarose drop test at 48 h. DS-MSCs migrated greater distances than the rest of the cell lines in both unstimulated conditions and in the presence of the inflammatory cytokines TNF-α and IL-1β (Fig. 7, a–c).

SCA-MSCs and DS-MSCs significantly increased their migration capacity in the presence of IL-1β compared to the control with PBS. Moreover, IL-1β was a significantly more potent stimulus than TNF-α for the AA-MSC and PB-MSC cell lines (Fig. 7).

## Discussion

The results of the present study clearly demonstrated that AA-MSCs, SCA-MSCs, DS-MSCs and PB-MSCs shared similar characteristics in terms of morphology, alkaline phosphatase activity, expression of cell surface and pluripotency-related markers, differentiation ability into adipocytes and proliferative capacity. In addition, all MSC lines analyzed showed in vitro migration ability of mesenchymal cells.

Our findings showed that porcine MSCs could be isolated from abdominal adipose, subcutaneous adipose, dermal skin and peripheral blood tissues from an adult male Iberian pig and successfully expanded in vitro. Passaged cells had more homogeneous morphology than primary cultures and formed colonies as the culture progressed. These morphological observations suggest that the isolated cells may contain both mature and progenitor populations as has been demonstrated in previous studies [35–37]. The use of MSCs in cell therapy involves in vitro expansion to achieve a sufficient number of cells, which implicitly carries the risk of propagating cells with genetic abnormalities during cell culture. Genetic abnormalities may lead to transformation and poor performance in clinical use, and are a critical safety concern for cell therapies using MSCs [38]. Karyotyping is a practical way to assess genome stability and can be useful as part of initial characterization of an MSC population. AA-MSCs expanded through 10 passages did not show chromosomal translocations, deletions or abnormal chromosome number.

Many studies demonstrate that the ability to express alkaline phosphatase activity is a pluripotency marker of stem cells including porcine MSCs from umbilical cord [39] and from skin [40]. However, many other authors do not yield such conclusive results, showing that alkaline phosphatase activity decreases with donor age regardless of the sex of the pig and tissue type [5]. On the other hand, the level of staining of cells expressing alkaline phosphatase activity is not always uniform, varying according to the tissue source studied [5]. There are also studies demonstrating that the expression of alkaline phosphatase varies over time during the assay [41]. Contradictory results have been obtained in studies of tissue-specific MSCs using alkaline phosphatase activity as a measure of stem cell maintenance capability [42]. Ock et al. [43] found that canine adipose MSCs have extremely low AP activity but have a higher potential for differentiation along the osteogenesis and adipogenesis pathways than do other MSC types. Consistent with this, Ock et al. [5] also found that porcine adipose MSCs were more capable of undergoing in vitro differentiation, also having the lowest AP activity. Our MSCs derived from all sources were positive for AP activity. The lowest level was observed in DS-MSCs. Similar results were shown by Song et al. [37], who reported a greater intensity of AP expression in MSCs of adipose origin, compared to MSCs from cutaneous origin.

Therefore, to confirm the multipotency of MSCs, we examined the expression of typical markers of multipotent mesenchymal stem cells reported in the literature. Major histocompatibility complex class II (MHCII) molecules are found in antigen-presenting cells such as dendritic cells, mononuclear phagocytes, some endothelial cells, thymic epithelial cells and B cells. The MHCII expression in MSC must be negative [34]. CD34 is an antigen of hematopoietic progenitor cells that should also be absent in MSCs, since these cells do not have hematopoietic characteristics [44]. Vimentin is the main component of the intermediate filament cytoskeleton of mesenchymal cells, involved in adhesion, migration and cell signaling. It is commonly used as a marker for mesenchymal cells and mesenchymal histopathological diagnosis, and has been previously used as a positive cell marker when characterizing porcine mesenchymal cells [34, 45]. CD44 is a cell adhesion surface molecule present in porcine MSCs as demonstrated in numerous studies of cell characterization the same as CD105 and CD90 [16, 45]. A disadvantage of CD105 is a limited cross-reactivity of anti-human antibodies with animal cells [46].

POU5F1 domain Oct-4 transcription factor has been considered one of the main regulators of differentiation and self-renewal of pluripotent stem cells [47]. It is important to note that the expression of POU5F1 can be studied at the level of the protein using western blot assay or immunostaining; or at the mRNA level by PCR amplification methods [48]. Recent studies have reported the detection of this transcription factor in porcine MSCs from umbilical cord, dermal skin, bone marrow and adipose and ovarian tissues [5, 35, 37, 49]. Most of the assays performed indicate that the expression of POU5F1 depends on the cell passage number, cell source and age [42, 50]. The expression of this marker is variable according to the source, reflecting the fact that some mesenchymal cells have greater capacity of stemness than others [5].

Our data demonstrate that MSCs derived from abdominal adipose, subcutaneous adipose, dermal skin and peripheral blood tissues were negative for cytokeratin (except DS-MSCs), MHCII and CD34, but positive for vimentin and POU5F1, and strongly positive for CD44. Expression of POU5F1 was confirmed by flow cytometry in dermal skin MSCs and bone marrow MSCs [5].

Previous studies showed that bone marrow, skin and adipose tissue-derived MSCs were positive for vimentin, but negative for cytokeratin [37]. However, in our analyses, although DS-MSCs were positive for vimentin, they also showed low levels of cytokeratin expression. Cytokeratin is also a component of intermediate filament cytoskeleton but is restricted to epithelial tissues. The expression of these cytokeratins is therefore specific to epithelial cells, making it a cellular marker used for the diagnosis and characterization of tissues. Song et al. [37] have also reported cytokeratin expression in porcine MSCs derived from adipose and ovarian tissue.

The ability of MSCs to divide and differentiate could be assessed, at least in part, by evaluating their proliferative capacity. One of the characteristics of mesenchymal cells is their almost unlimited proliferation capacity [34]. Studies show that the proliferative and self-renewing capacity of this type of cells is related to telomerase activity and expression of OCT3/4 [51]. Some reports show that the proliferative capacity of porcine mesenchymal cells decrease as the age of the donor animal increases [52]. Likewise, this property is different according to the type of tissue studied, so that differences between the proliferation rate in mesenchymal cells derived from bone marrow and adipose tissue have been reported [5]. It is important to highlight that in some cases MSCs are able to divide, but to a limited extent, in vitro before entering replicative senescence. Between passages 7 and 12, MSCs increase their cell size and reduce the expression of certain pluripotency markers, leading to proliferative arrest [53, 54]. However, it should also be considered that this event has not been demonstrated in MSCs of all species. All our mesenchymal lines were established from tissue samples of a single adult (2-year-old) Iberian pig and our results indicated that DS-MSCs had the greatest proliferation potential while AA-MSCs showed the longest population doubling time. In addition, all MSC lines had high proliferative capacity until passages 9–11 as shown in the proliferation assay. At that time, robust proliferation was always observed. In this regard, Li et al. [55] reported a novel role for vimentin, highly expressed in our cells, in connection with AFP^+^ cells and BrdU^+^ cells, indicating that these cells are activated for proliferation.

Multipotent differentiation potential is one of the defined criteria proposed by the ISCT, making MSCs a favorable choice in regenerative therapy [12]. MSCs have a unique quality of multilineage differentiation upon induction with specific differentiation media, supplemented with growth factors. Understanding the molecular mechanism, intracellular pathways and factors responsible for various differentiation abilities of MSCs from different sources has been a matter of great interest in the last decades. Initial investigations were mainly focused on mesodermal differentiation capacities of stem cells; however, with advances in knowledge and technology such as gene targeting and protein engineering, MSC research has reached beyond mesodermal differentiation to multilineage specialized cell differentiation, revolutionizing the field of regenerative medicine. Our data for AA-MSCs, SCA-MSCs, DS-MSCs and PB-MSCs revealed the basic in vitro trilineage differentiation capacity that is adipocytes, osteocytes and chondrocytes, as observed previously in the swine model [56–58] and human MSCs [59, 60].

One of the most remarkable findings is the ability of MSCs to migrate from bone marrow or peripheral blood into damaged tissues. MSC are currently being investigated for use in a wide variety of clinical applications. For most of these applications, systemic delivery of the cells is preferred. However, this requires the homing and migration of MSCs to a target tissue. Recently, Almalki et al. [24] reported the migratory activity of porcine AA-MSCs and evaluated the effect of MMP-2, MMP-14 and ATR2 siRNA silencing in this cell line migration. Our results indicated that all MSC lines showed migration activity. The observed nonchemotactic invasion into PBS-containing spots is most likely due to the highly motile nature of these MSC lines. Accordingly, DS-MSCs migrated greater distances than the rest of the cell lines both in the absence or the presence of the inflammatory cytokines TNF-α and IL-1β. SCA-MSCs and DS-MSCs significantly increased their migration capacity in the presence of IL-1β after 48 h compared to the control in PBS.

The literature has reported that MSCs exhibit both tissue and donor-related variability, not only in mRNA expression but also with regard to chemokine and cytokine production [61–65]. Future studies will aim at analyzing the degree of individual variability presented by the different MSCs isolated in this work.

This report shows for the first time a comparative study of porcine MSCs of different tissue origin, including PB-MSCs. To date, porcine PB-MSCs have only been compared to bone marrow MSCs [30, 66] and AA-MSCs [67]. The migration capacity of porcine AA-MSCs has recently been reported [24], but a comparative study of migration capacity between different lines of porcine MSCs is shown here for the first time.

## Conclusions

In summary, this study describes the isolation and characterization of porcine cell lines from different tissue origin, with a clear mesenchymal pattern. We show for the first time a comparative study including the migration capacity induced by inflammatory mediators of porcine MSCs of different tissue origin.

## Abbreviations

AA-MSC: Abdominal adipose tissue mesenchymal stem/stromal cell
BSA: Bovine serum albumin
DMEM-LG: Dulbecco’s modified Eagle’s medium low glucose
DS-MSC: Dermal skin tissue mesenchymal stem/stromal cell
HBSS: Hank’s Balanced Salt Solution
IL-1β: Interleukin-1β
MHCII: Major histocompatibility complex II
MSC: Mesenchymal stem/stromal cell
PB-MSC: Peripheral blood mesenchymal stem/stromal cell
POU5F1: POU class 5 homeobox 1
SCA-MSC: Subcutaneous adipose tissue mesenchymal stem/stromal cell
T/E: Trypsin–ethylenediamine tetraacetic acid
TNF-α: Tumor necrosis factor alpha

## References

[1] Friedenstein AJ, Gorskaja JF, Kulagina NN (1976). Fibroblast precursors in normal and irradiated mouse hematopoietic organs. Exp Hematol.

[2] Lindner U, Kramer J, Rohwedel J, Schlenke P (2010). Mesenchymal stem or stromal cells: toward a better understanding of their biology. Transfus Med Hemother.

[3] Caplan AI (2017). Mesenchymal stem cells: time to change the name. Stem Cells Transl Med.

[4] Trohatou O, Roubelakis MG (2017). Mesenchymal stem/stromal cells in regenerative medicine: past, present, and future. Cell Reprogram.

[5] Ock SA, Baregundi Subbarao R, Lee YM, Lee JH, Jeon RH, Lee SL (2016). Comparison of immunomodulation properties of porcine mesenchymal stromal/stem cells derived from the bone marrow, adipose tissue, and dermal skin tissue. Stem Cells Int.

[6] Carrade DD, Lame MW, Kent MS, Clark KC, Walker NJ, Borjesson DL (2012). Comparative analysis of the immunomodulatory properties of equine adult-derived mesenchymal stem cells. Cell Med.

[7] Fu WL, Li J, Chen G, Li Q, Tang X, Zhang CH (2015). Mesenchymal stem cells derived from peripheral blood retain their pluripotency, but undergo senescence during long-term culture. Tissue Eng Part C Methods.

[8] Uccelli A, Pistoia V, Moretta L (2007). Mesenchymal stem cells: a new strategy for immunosuppression. Trends Immunol.

[9] Parys M, Kruger JM, Yuzbasiyan-Gurkan V (2017). Evaluation of immunomodulatory properties of feline mesenchymal stem cells. Stem Cells Dev.

[10] Chow L, Johnson V, Coy J, Regan D, Dow S (2017). Mechanisms of immune suppression utilized by canine adipose and bone marrow-derived mesenchymal stem cells. Stem Cells Dev.

[11] Gallardo D, de la Cámara R, Nieto JB, Espigado I, Iriondo A, Jiménez-Velasco A (2009). Is mobilized peripheral blood comparable with bone marrow as a source of hematopoietic stem cells for allogeneic transplantation from HLA-identical sibling donors? A case-control study. Haematologica.

[12] Dominici M, Le Blanc K, Mueller I, Slaper-Cortenbach I, Marini F, Krause D (2006). Minimal criteria for defining multipotent mesenchymal stromal cells. The International Society for Cellular Therapy position statement. Cytotherapy.

[13] Squillaro T, Peluso G, Galderisi U (2016). Clinical trials with mesenchymal stem cells: an update. Cell Transplant.

[14] Swindle MM, Makin A, Herron AJ, Clubb FJ, Frazier KS (2012). Swine as models in biomedical research and toxicology testing. Vet Pathol.

[15] Ringe J, Kaps C, Burmester GR, Sittinger M (2002). Stem cells for regenerative medicine: advances in the engineering of tissues and organs. Naturwissenschaften.

[16] Ramírez O, Burgos-Paz W, Casas E, Ballester M, Bianco E, Olalde I (2015). Genome data from a sixteenth century pig illuminate modern breed relationships. Heredity (Edinb).

[17] Gonzalez-Bulnes A, Astiz S, Ovilo C, Lopez-Bote CJ, Torres-Rovira L, Barbero A (2016). Developmental origins of health and disease in swine: implications for animal production and biomedical research. Theriogenology.

[18] Benítez R, Fernández A, Isabel B, Núñez Y, De Mercado E, Gómez-Izquierdo E, et al. Modulatory effects of breed, feeding status, and diet on adipogenic, lipogenic, and lipolytic gene expression in growing Iberian and Duroc pigs. Int J Mol Sci. 2017;19:22. 10.3390/ijms19010022 PMC5795973 29271889

[19] Torres-Rovira L, Astiz S, Caro A, Lopez-Bote C, Ovilo C, Pallares P (2012). Diet-induced swine model with obesity/leptin resistance for the study of metabolic syndrome and type 2 diabetes. ScientificWorldJournal.

[20] Mackenzie TC, Flake AW (2001). Human mesenchymal stem cells persist, demonstrate site-specific multipotential differentiation, and are present in sites of wound healing and tissue regeneration after transplantation into fetal sheep. Blood Cells Mol Dis.

[21] Kawada H, Fujita J, Kinjo K, Matsuzaki Y, Tsuma M, Miyatake H (2004). Nonhematopoietic mesenchymal stem cells can be mobilized and differentiate into cardiomyocytes after myocardial infarction. Blood.

[22] Koç ON, Gerson SL, Cooper BW, Dyhouse SM, Haynesworth SE, Caplan AI (2000). Rapid hematopoietic recovery after coinfusion of autologous-blood stem cells and culture-expanded marrow mesenchymal stem cells in advanced breast cancer patients receiving high-dose chemotherapy. J Clin Oncol.

[23] Horwitz EM, Prockop DJ, Fitzpatrick LA, Koo WW, Gordon PL, Neel M (1999). Transplantability and therapeutic effects of bone marrow-derived mesenchymal cells in children with osteogenesis imperfecta. Nat Med.

[24] Almalki SG, Agrawal DK (2017). ERK signaling is required for VEGF-A/VEGFR2-induced differentiation of porcine adipose-derived mesenchymal stem cells into endothelial cells. Stem Cell Res Ther.

[25] Roufosse CA, Direkze NC, Otto WR, Wright NA (2004). Circulating mesenchymal stem cells. Int J Biochem Cell Biol.

[26] Lyahyai J, Mediano DR, Ranera B, Sanz A, Remacha AR, Bolea R (2012). Isolation and characterization of ovine mesenchymal stem cells derived from peripheral blood. BMC Vet Res.

[27] Spaas JH, De Schauwer C, Cornillie P, Meyer E, Van Soom A, Van de Walle GR (2013). Culture and characterisation of equine peripheral blood mesenchymal stromal cells. Vet J.

[28] Sato K, Yamawaki-Ogata A, Kanemoto I, Usui A, Narita Y (2016). Isolation and characterisation of peripheral blood-derived feline mesenchymal stem cells. Vet J.

[29] Fu Q, Zhang Q, Jia LY, Fang N, Chen L, Yu LM, et al. Isolation and characterization of rat mesenchymal stem cells derived from granulocyte Colony-stimulating factor-mobilized peripheral blood. Cells Tissues Organs. 2015-16;201:412-22. 10.1159/000445855 27246344

[30] Faast R, Harrison SJ, Beebe LF, McIlfatrick SM, Ashman RJ, Nottle MB (2006). Use of adult mesenchymal stem cells isolated from bone marrow and blood for somatic cell nuclear transfer in pigs. Cloning Stem Cells.

[31] Rodríguez A, Sanz E, De Mercado E, Gómez E, Martín M, Carrascosa C (2010). Reproductive consequences of a reciprocal chromosomal translocation in two Duroc boars used to provide semen for artificial insemination. Theriogenology.

[32] Wiggins H, Rappoport J (2010). An agarose spot assay for chemotactic invasion. BioTechniques.

[33] Miyamoto Y, Skarzynski DJ (2000). Okuda K. Is tumor necrosis factor alpha a trigger for the initiation of endometrial prostaglandin F(2alpha) release at luteolysis in cattle. Biol Reprod.

[34] Bharti D, Shivakumar SB, Subbarao RB, Rho GJ (2016). Research advancements in porcine derived mesenchymal stem cells. Curr Stem Cell Res Ther.

[35] Kang EJ, Byun JH, Choi YJ, Maeng GH, Lee SL, Kang DH (2010). In vitro and in vivo osteogenesis of porcine skin-derived mesenchymal stem cell-like cells with a demineralized bone and fibrin glue scaffold. Tissue Eng Part A.

[36] Williams KJ, Picou AA, Kish SL, Giraldo AM, Godke RA, Bondioli KR (2008). Isolation and characterization of porcine adipose tissue-derived adult stem cells. Cells Tissues Organs.

[37] Song S-H, Kumar BM, Kang E-J, Lee Y-M, Kim T-H, Ock S-A (2011). Characterization of porcine multipotent stem/stromal cells derived from skin, adipose, and ovarian tissues and their differentiation in vitro into putative oocyte-like cells. Stem Cells Dev.

[38] Stultz BG, McGinnis K, Thompson EE, Lo Surdo JL, Bauer SR, Hursh DA (2016). Chromosomal stability of mesenchymal stromal cells during in vitro culture. Cytotherapy.

[39] Carlin R, Davis D, Weiss M, Schultz B, Troyer D (2006). Expression of early transcription factors Oct-4, Sox-2 and Nanog by porcine umbilical cord (PUC) matrix cells. Reprod Biol Endocrinol.

[40] Kumar BM, Yoo JG, Ock SA, Kim JG, Song HJ, Kang EJ (2007). In vitro differentiation of mesenchymal progenitor cells derived from porcine umbilical cord blood. Mol Cells.

[41] Juhásová J, Juhás S, Klíma J, Strnádel J, Holubová M, Motlík J (2011). Osteogenic differentiation of miniature pig mesenchymal stem cells in 2D and 3D environment. Physiol Res.

[42] Chen J, Lu Z, Cheng D, Peng S, Wang H (2011). Isolation and characterization of porcine amniotic fluid-derived multipotent stem cells. PLoS One.

[43] Ock SA, Maeng GH, Lee YM, Kim TH, Kumar BM, Lee SL (2013). Donor-matched functional and molecular characterization of canine mesenchymal stem cells derived from different origins. Cell Transplant.

[44] Wang X, Zheng F, Liu O, Zheng S, Liu Y, Wang Y (2013). Epidermal growth factor can optimize a serum-free culture system for bone marrow stem cell proliferation in a miniature pig model. In Vitro Cell Dev Biol Anim..

[45] Park BW, Kang DH, Kang EJ, Byun JH, Lee JS, Maeng GH (2012). Peripheral nerve regeneration using autologous porcine skin-derived mesenchymal stem cells. J Tissue Eng Regen Med.

[46] Boxall SA, Jones E (2012). Markers for characterization of bone marrow multipotential stromal cells. Stem Cells Int..

[47] Kashyap V, Rezende NC, Scotland KB, Shaffer SM, Persson JL, Gudas LJ (2009). Regulation of stem cell pluripotency and differentiation involves a mutual regulatory circuit of the NANOG, OCT4, and SOX2 pluripotency transcription factors with polycomb repressive complexes and stem cell microRNAs. Stem Cells Dev.

[48] Subbarao RB, Ullah I, Kim EJ, Jang SJ, Lee WJ, Jeon RH (2015). Characterization and evaluation of neuronal trans-differentiation with electrophysiological properties of mesenchymal stem cells isolated from porcine endometrium. Int J Mol Sci.

[49] Kang EJ, Lee YH, Kim MJ, Lee YM, Kumar BM, Jeon BG, et al. Transplantation of porcine umbilical cord matrix mesenchymal stem cells in a mouse model of Parkinson’s disease. J Tissue Eng Regen Med. 2013;7:169-82. 10.1002/term.504 22081626

[50] Ock SA, Jeon BG, Rho GJ (2010). Comparative characterization of porcine mesenchymal stem cells derived from bone marrow extract and skin tissues. Tissue Eng Part C Methods..

[51] Simonsen JL, Rosada C, Serakinci N, Justesen J, Stenderup K, Rattan SI (2002). Telomerase expression extends the proliferative life-span and maintains the osteogenic potential of human bone marrow stromal cells. Nat Biotechnol.

[52] Rando TA (2006). Stem cells, ageing and the quest for immortality. Nature.

[53] Wagner W, Horn P, Castoldi M, Diehlmann A, Bork S, Saffrich R (2008). Replicative senescence of mesenchymal stem cells: a continuous and organized process. PLoS One.

[54] Alessio N, Del Gaudio S, Capasso S, Di Bernardo G, Cappabianca S, Cipollaro M (2015). Low dose radiation induced senescence of human mesenchymal stromal cells and impaired the autophagy process. Oncotarget.

[55] Li B, Zheng YW, Sano Y, Taniguchi H (2011). Evidence for mesenchymal-epithelial transition associated with mouse hepatic stem cell differentiation. PLoS One.

[56] Dariolli R, Bassaneze V, Nakamuta JS, Omae SV, Campos LC, Krieger JE (2013). Porcine adipose tissue-derived mesenchymal stem cells retain their proliferative characteristics, senescence, karyotype and plasticity after long-term cryopreservation. PLoS One.

[57] Qu CQ, Zhang GH, Zhang LJ, Yang GS (2007). Osteogenic and adipogenic potential of porcine adipose mesenchymal stem cells. In Vitro Cell Dev Biol Anim.

[58] Arrigoni E, Lopa S, de Girolamo L, Stanco D, Brini AT (2009). Isolation, characterization and osteogenic differentiation of adipose-derived stem cells: from small to large animal models. Cell Tissue Res.

[59] Zuk PA, Zhu M, Ashjian P, De Ugarte DA, Huang JI, Mizuno H (2002). Human adipose tissue is a source of multipotent stem cells. Mol Biol Cell.

[60] Blande IS, Bassaneze V, Lavini-Ramos C, Fae KC, Kalil J, Miyakawa AA (2009). Adipose tissue mesenchymal stem cell expansion in animal serum-free medium supplemented with autologous human platelet lysate. Transfusion.

[61] Zhukareva V, Obrocka M, Houle JD, Fischer I, Neuhuber B (2010). Secretion profile of human bone marrow stromal cells: donor variability and response to inflammatory stimuli. Cytokine.

[62] Paradisi M, Alviano F, Pirondi S, Lanzoni G, Fernandez M, Lizzo G (2014). Human mesenchymal stem cells produce bioactive neurotrophic factors: source, individual variability and differentiation issues. Int J Immunopathol Pharmacol.

[63] Vakhrushev IV, Vdovin AS, Strukova LA, Yarygin KN (2016). Variability of the phenotype and proliferation and migration characteristics of human mesenchymal stromal cells derived from the deciduous teeth pulp of different donors. Bull Exp Biol Med.

[64] Lavoie JR, Creskey MM, Muradia G, Bell GI, Sherman SE, Gao J (2016). Brief report: elastin microfibril Interface 1 and integrin-linked protein kinase are novel markers of islet regenerative function in human multipotent mesenchymal stromal cells. Stem Cells.

[65] Paladino FV, Sardinha LR, Piccinato CA, Goldberg AC (2017). Intrinsic variability present in Wharton’s jelly mesenchymal stem cells and T cell responses may impact cell therapy. Stem Cells Int.

[66] Heino TJ, Alm JJ, Moritz N, Aro HT (2012). Comparison of the osteogenic capacity of minipig and human bone marrow-derived mesenchymal stem cells. J Orthop Res.

[67] Yang Z, Vajta G, Xu Y, Luan J, Lin M, Liu C (2016). Production of pigs by hand-made cloning using mesenchymal stem cells and fibroblasts. Cell Reprogram..

